# The Perils of a “My Work Here is Done” perspective: a mixed methods evaluation of sustainment of an evidence-based intervention for transient ischemic attack

**DOI:** 10.1186/s12913-022-08207-8

**Published:** 2022-07-04

**Authors:** Dawn M. Bravata, Edward J. Miech, Laura J. Myers, Anthony J. Perkins, Ying Zhang, Nicholas A. Rattray, Sean A. Baird, Lauren S. Penney, Curt Austin, Teresa M. Damush

**Affiliations:** 1 Department of Veterans Affairs (VA) Health Services Research and Development (HSR&D) Precision Monitoring to Transform Care (PRISM) Quality Enhancement Research Initiative (QUERI), Indianapolis, IN USA; 2 VA HSR&D Center for Health Information and Communication (CHIC), Richard L. Roudebush VA Medical Center, HSR&D Mail Code 11H, 1481 West 10th Street, Indianapolis, IN 46202 USA; 3 Department of Internal Medicine, Indiana University School of Medicine, Indianapolis, IN USA; 4 Department of Neurology, Indiana University School of Medicine, Indianapolis, IN USA; 5 Regenstrief Institute, Indianapolis, IN USA; 6 Department of Biostatistics, Indiana University School of Medicine, IN Indianapolis, USA; 7 Department of Biostatistics, College of Public Health, University of Nebraska Medical Center, Omaha, NE USA; 8 VA HSR&D Elizabeth Dole Center of Excellence for Veteran and Caregiver Research, South Texas Veterans Health Care System, San Antonio, TX USA; 9 Department of Medicine, University of Texas Health San Antonio, San Antonio, TX USA

**Keywords:** Sustainment, Quality of care, Implementation science, Cerebrovascular disease

## Abstract

**Background:**

To evaluate quality improvement sustainment for Transient Ischemic Attack (TIA) and identify factors influencing sustainment, which is a challenge for Learning Healthcare Systems.

**Methods:**

Mixed methods were used to assess changes in care quality across periods (baseline, implementation, sustainment) and identify factors promoting or hindering sustainment of care quality. PREVENT was a stepped-wedge trial at six US Department of Veterans Affairs implementation sites and 36 control sites (August 2015—September 2019). Quality of care was measured by the without-fail rate: proportion of TIA patients who received all of the care for which they were eligible among brain imaging, carotid artery imaging, neurology consultation, hypertension control, anticoagulation for atrial fibrillation, antithrombotics, and high/moderate potency statins. Key informant interviews were used to identify factors associated with sustainment.

**Results:**

The without-fail rate at PREVENT sites improved from 36.7% (baseline, 58/158) to 54.0% (implementation, 95/176) and settled at 48.3% (sustainment, 56/116). At control sites, the without-fail rate improved from 38.6% (baseline, 345/893) to 41.8% (implementation, 363/869) and remained at 43.0% (sustainment, 293/681). After adjustment, no statistically significant difference in sustainment quality between intervention and control sites was identified. Among PREVENT facilities, the without-fail rate improved ≥2% at 3 sites, declined ≥2% at two sites, and remained unchanged at one site during sustainment. Factors promoting sustainment were planning, motivation to sustain, integration of processes into routine practice, leadership engagement, and establishing systems for reflecting and evaluating on performance data. The only factor that was sufficient for improving quality of care during sustainment was the presence of a champion with plans for sustainment. Challenges during sustainment included competing demands, low volume, and potential problems with medical coding impairing use of performance data. Four factors were sufficient for declining quality of care during sustainment: low motivation, champion inactivity, no reflecting and evaluating on performance data, and absence of leadership engagement.

**Conclusions:**

Although the intervention improved care quality during implementation; performance during sustainment was heterogeneous across intervention sites and not different from control sites. Learning Healthcare Systems seeking to sustain evidence-based practices should embed processes within routine care and establish systems for reviewing and reflecting upon performance.

**Trial registration:**

Clinicaltrials.gov (NCT02769338)

**Supplementary Information:**

The online version contains supplementary material available at 10.1186/s12913-022-08207-8.

## CALLOUT BOX

### What is known on this topic?

- Sustaining quality improvement has been recognized as a key implementation challenge for healthcare systems.
- Sustainment is a key element of the Learning Healthcare System model which seeks to promote continuous improvement.

### What this study adds?

- Although the intervention improved care quality during implementation; performance during sustainment was heterogeneous across intervention sites and not different from control sites.
- This study identified factors associated with sustainment after achieving implementation success across diverse medical centers: planning, motivation to sustain, embedding key processes of care into routine practice, leadership engagement, and establishing systems for reflecting and evaluating on performance data to plan quality improvement activities or respond to changes in quality.
- The use of matched control sites provided a context for interpreting changes in facility performance over time.

## Introduction

Many studies, across disciplines, have described projects which initially enjoyed quality improvement success, but which were not able to sustain quality improvement over time [1–3]. Sustainment involves ingraining processes that were successful during active implementation into routine workflow and hence ongoing facilitation is no longer required to maintain quality improvement [4–6]. Sustainment has been recognized as a key problem in quality improvement, with an estimated 70% of change initiatives not being sustained [6, 7].

Sustainment is a key element of the Learning Healthcare System model [8, 9]. The US Institute of Medicine’s described the Learning Healthcare System as an approach where “clinical informatics, incentives, and culture are aligned to promote continuous improvement and innovation, with best practices seamlessly embedded in the delivery process and new knowledge captured as an integral by-product of the delivery experience” [10]. Sites that have implemented Learning Healthcare System models are ones that continuously learn from quality improvement activities about interventions which either do or do not work within the site-specific context [11]; they progress from performance measurement to a deep understanding of the strengths and weaknesses of their system [11]. During sustainment, Learning Healthcare Systems demonstrate the value of evidence-based interventions to stakeholders and transition interventions to leadership for ongoing maintenance without external support [9]. The growing literature about Learning Healthcare Systems has described a variety of interventions that promote quality improvement, however, less is known about effective strategies to sustain quality improvement across healthcare systems [12, 13]. Similarly, the National Health Service Sustainability Model has identified factors that are associated with sustainment (e.g., characteristics of the monitoring system, staff involvement, leadership engagement), but evidence-based approaches to overcoming sustainment barriers are lacking [6]. Moreover, little is known about the contextual factors related to evidence-based sustainment [6].

The Protocol-Guided Rapid Evaluation of Veterans Experiencing New Transient Neurological Symptoms (PREVENT) quality improvement program was designed in alignment with the Learning Healthcare System model to improve quality of care for patients with transient ischemic attack (TIA) [14, 15]. The objectives of this sustainment evaluation were to: (1) examine the degree to which improvement in the delivery of guideline-concordant processes of care was sustained after the end of active implementation; and (2) identify contextual factors contributing to sustainment. This manuscript adheres both to the Standards for Reporting Implementation Studies (StaRI) statement [16] and the STrengthening the Reporting of OBservational studies in Epidemiology (STROBE) guidelines [17].

## Methods

### Overview of sustainment evaluation

Sustainment was conceptualized as the maintenance of quality of care (which is the result of quality improvement activities such as plan-do-study-act [18] projects). Quality of care was assessed quantitatively by the without-fail rate (defined below) across three study periods: baseline, active implementation, sustainment. Factors that were associated with sustainment of quality of care was assessed qualitatively. This sustainment evaluation was a pre-planned component of a five-year nonrandomized augmented stepped-wedge trial at six sites where active implementation was initiated in three waves, with two facilities per wave [19, 20].

#### Sites

The PREVENT study has been described previously [15, 21–25]. A total of 42 Veterans Health Administration (VA) facilities were included in the study: 6 were PREVENT active implementation sites and 36 were usual care control sites. The six active implementation sites were each matched to six control sites on the basis of baseline facility characteristics: TIA patient volume, facility complexity (e.g., teaching status, intensive care unit level), and quality of care (measured by the without-fail rate, described below).

#### Context

The VA is the largest integrated healthcare system in the United States. In the VA, quality measurement is integrated into the healthcare system administration and clinical operations [26, 27]. Although stroke care quality metrics are reported, there was no VA system-wide focus on TIA care quality. None of six participating PREVENT facilities were engaged in TIA-focused quality improvement at baseline. All six sites had access to all 7 of the clinical processes targeted in PREVENT.

### Study periods

The one-year baseline period was defined as the 12-months prior to the baseline site visit at each participating facility (August 21, 2015—January 30, 2018). The one-year active implementation period began one month after the site’s kick-off (August 11, 2017—May 12, 2019). The sustainment period immediately followed active implementation and ended for all sites on September 30, 2019 (August 11, 2018—September 30, 2019). The sustainment period varied in duration with Wave-1 sites having a longer sustainment than Wave-3 sites (range: 4.5—13.7 months). The definition (i.e., the dates) of the study periods (baseline, active implementation, and sustainment) for each control site was identical to the definition used for the matched PREVENT site.

### Quality Improvement Intervention

The PREVENT quality improvement intervention consisted of five components: clinical programs, data feedback, professional education, electronic health record tools, and quality improvement support including a virtual collaborative [15]. The intervention targeted facility clinical staff. The composition of the facility teams varied [22], but generally included neurology, emergency medicine, nursing, pharmacy and radiology. Some teams also included hospitalists, primary care providers, education staff, telehealth staff, or systems redesign staff [22]. The clinical champions at each site were diverse; the majority were neurologists, but an Emergency Department nurse and a systems redesign staff member were also champions [22].

Active implementation began with a kickoff during which the facility team: explored their local performance data; identified barriers to providing highest quality of care; described potential solutions to address barriers; and developed a site-specific action plan. The PREVENT web-based hub provided process and outcome data, allowing teams to interact with their site’s performance data to explore hypotheses and monitor performance over time [23]. The teams joined monthly virtual collaborative conferences during which the teams shared progress on action plans, articulated goals for the next month, and reviewed new evidence or tools [25]. Sustainment was explicitly included as a topic during three of the monthly collaborative calls including discussions about how to incorporate key processes into existing structures and the value of engaging with performance data for reflecting and evaluating, goal setting with feedback, and planning.

#### External Facilitation: The Primary Difference between Active Implementation and Sustainment

The implementation and evaluation of the PREVENT quality improvement intervention were guided by the Consolidated Framework for Implementation Research (CFIR) [28]. PREVENT employed: team activation via audit and feedback, reflecting and evaluating, planning, and goal setting; external facilitation; and building a community of practice [23, 25].

External facilitation was one of the bundled implementation strategies provided by the coordinating center team to the participating site team members principally by a nurse with experience in quality improvement and a physician with experience in cerebrovascular disease and quality improvement during 12 months of active implementation. The external facilitation nurse supported action planning, fostered quality improvement skills, and promoted the practice of reflecting and evaluating on performance data. External facilitation was performed primarily by telephone and email but also occurred during regularly scheduled office hours.

The key difference between active implementation and sustainment was that external facilitation was no longer initiated by the coordinating center study team [24]. For example, if the study team observed a decrement in a participating site’s care quality during active implementation, the external facilitator would reach out to the site to offer support and encouragement. However, during sustainment, facilitation was only provided in response to explicit requests by the participating site’s team members. For example, the facilitator would answer questions about individual cases and take the opportunity to remind the requestor to visit the data hub to review site-level data and shared resources. During the sustainment period the site team members were able to attend the monthly collaborative conferences [29]; and they had full access to the PREVENT hub which provided their facility’s updated performance data [23].

### Mixed methods assessment of sustainment

We employed a convergent parallel, mixed-methods design to evaluate sustainment with prospective data collection from multiple sources. We began with quantitative analyses to identify sites which either did or did not sustain quality of care. Then we used qualitative analyses to identify contextual factors associated with sustainment.

#### Quantitative analysis of sustained quality of care

##### Quality of care outcome measures

The primary measure of quality of care was the facility-level “without-fail” rate, which was an “all-or-none” measure of quality of care. It was defined as the proportion of Veterans with TIA at a specific facility, who received all of the processes of care for which they were eligible from among seven processes of care: brain imaging, carotid artery imaging, neurology consultation, hypertension control, anticoagulation for atrial fibrillation, antithrombotics, and high/moderate potency statins [15, 21, 30]. The numerator and denominator definitions for each of the seven processes of care have been previously described [21]. The without-fail rate was calculated for each site over the three study periods (i.e., baseline, active implementation, sustainment). The without-fail rate was based on guideline recommended processes of care [31, 32]; it has been associated with improved patient outcomes [33].

Given the all-or-none nature of the without-fail rate, it can be a relatively difficult to change and even small improvements in the absolute rate may reflect substantial changes in facility’s practice [34]. The without-fail rate was either zero or one for each patient (they either did or did not receive all of the care for which they were eligible), but varied theoretically from 0-100% at the facility level. Sites with difficulty providing several processes (e.g., carotid imaging and blood pressure control) had greater quality improvement challenges than sites which had only opportunities for improvement in one process (e.g., high/moderate potency statins).

The secondary quality outcomes were the individual process measures (each defined as the number of patients who received a process divided by number of patients who were eligible for a process) as well as the facility-level consolidated measure of quality which described the number of patients who received any of the seven processes (“passes”) divided by number of patients who were eligible for processes (“opportunities”).

##### Classifying sites in terms of quality of care sustainment

The six PREVENT active implementation sites were classified as improving, declining, or not changing in terms of the absolute change in the without-fail rate over study periods, where a change of ≥2% in the without-fail rate was considered as a meaningful change in quality of care [34]. This site classification was used in the qualitative analyses (described below) to identify factors that either promoted or hindered sustainment of care quality.

##### Data sources

We retrospectively identified Veteran patients with TIA who were cared for in the Emergency Department or inpatient setting [15] based on primary discharge codes [15, 30]. The processes of care were assessed with electronic health record data using validated algorithms [30, 35]. Process of care data were obtained from the VA Corporate Data Warehouse which included: inpatient and outpatient data files, healthcare utilization, and receipt of procedures (Current Procedural Terminology [CPT], Healthcare Common Procedures Coding System, and International Classification of Disease (*ICD*-9 and *ICD*-10) procedure codes). Corporate Data Warehouse data were also used for vital signs, laboratory data, allergies, imaging, orders, medications, and consults. Fee-Basis Data were also used to identify inpatient and outpatient healthcare utilization and medical history from non-VA sources. For each patient, the data were pulled for the five years prior to the index TIA event through 90-days post-discharge [30].

##### Quantitative analyses

The primary quantitative analysis compared the average without-fail rate during sustainment to the without-fail rates during the baseline and active implementation periods among the PREVENT implementation sites adjusting for wave and site variations [21, 36]. We compared the change in the without-fail rate between the PREVENT sites and matched control sites to ameliorate the potential influence of temporal trends in care that may confound the assessment of the intervention effect within the stepped-wedge design [21]. Secondary analyses included an assessment of sustainment for the consolidated measures of quality and the seven individual processes of care [21]. In sensitivity analyses, we examined the quality metrics after excluding the Wave-3 site with the shortest sustainment duration. Fisher’s exact test was used to compare whether categorical variables differed between the PREVENT sites and matched control sites as well as between study periods (baseline, active implementation, sustainment) [21]. Two-sample t-tests or Wilcoxon Rank Sum tests were used to test whether continuous outcomes differed between the PREVENT intervention and matched control sites as well as between periods [21]. Generalized mixed-effects models with random effects for site and fixed effects for wave and period, were used to analyze the PREVENT intervention effects [37]. Separate risk adjustment models were constructed for each process of care, for the without-fail rate, and for the consolidated measure [21]. Fully risk adjusted models included site, wave, and the patient characteristics that were associated with the particular outcome of interest (e.g., the without-fail rate) [21]. The risk-adjusted models are presented in Supplemental Tables D-L. All analyses were performed using SAS Enterprise Guide version 7.11. The study was registered with clinicaltrials.gov (NCT02769338) and received human subjects approval from the Indiana University School of Medicine Institutional Review Board (IRB).

##### Qualitative analysis to identify factors associated with sustained quality of care

For the sustainment analysis, qualitative data included: (1) semi-structured, key informant interviews from 12-months after active implementation began and end of sustainment period; (2) observations and field notes from the site visits, as well as team debriefings after site visits and monthly collaborative conferences; and (3) Fast Analysis and Synthesis Template (FAST) facilitation tracking of interactions (e.g., emails) that occurred outside of planned interviews and collaborative calls [38, 39]. In addition, attendance was tracked at each collaborative call. Interviews were conducted (by TD, EM, LP, SB) in-person or by telephone to evaluate challenges and facilitators to PREVENT implementation, local adaptation, reach within the site, sustainment, motivation, and local context. Please see the Appendix for the Sustainment Interview Guide. Key informants included staff involved in the delivery of TIA care, their managers, and facility leadership; we also accepted “snowball” referrals from stakeholders. Upon receipt of verbal consent, interviews were audio-recorded. The audio-recordings were transcribed verbatim. Transcripts were de-identified and imported into Nvivo12 for data coding and analysis. Quotations provided in the results are labeled with the timing of the interview (e.g., at the end of the 12-month active implementation period which was the start of sustainment or at the end of sustainment], the site (which aligns with the table site labels), and an indicator for individual staff members.

The developed common codebook was both deductive and inductive; it included both *a priori* codes as well as codes that emerged from the data. We started with key CFIR constructs and implementation strategies (e.g., external facilitation) and then allowed for inductive themes to emerge. The qualitative analysis team coded the same initial set of transcripts to identify emergent themes, discuss and resolve discrepancies until consensus was reached, and finalize the codebook. Team meetings addressed emergent coding questions and the common codebook was updated to reflect team coding decisions.

Two team members independently coded transcripts using both the qualitative codebook. They also scored four Consolidated Framework for Implementation Research (CFIR) constructs (i.e., Goals & Feedback, Planning, Reflecting & Evaluating, and Champions) for both magnitude and valence on a scale of +2 (strong positive) to -2 (strong negative) in terms of influencing the implementation of PREVENT at that site. Each of the paired coded transcripts were merged as one file and reconciled using coding stripes in the software to identify discrepant codes. During reconciliation meetings, discrepant codes were discussed until consensus was reached; final codes were added to the NVivo database.

We conducted thematic qualitative analysis [40] across the sites and participants followed by comparative case analyses [41] where each of the six PREVENT sites represented a case. We compared cases for contextual factors associated with PREVENT sustainment.

## Results

The six PREVENT sites were geographically diverse including facilities in the West, Northeast, Southeast, and Midwest. The annual TIA patient volume varied across PREVENT and controls sites and across periods, ranging from 10 to 32 during sustainment (Table 1).

**Table 1:** Without-fail Rates at PREVENT Facilities over study periods

Facility	Without-fail Rate^**a**^			Sustainment Classification^**b**^	***P***-value		
	Baseline Period % (n/N)	Implementation Period % (n/N)	Sustainment Period % (n/N)		Sustainment vs. Baseline	Sustainment vs Implementation	Implementation vs Baseline
A	16.3 (7/43)	34.5 (10/29)	34.4 (11/32)	No change	0.101	1.000	0.094
B	33.3 (6/18)	44.4 (12/27)	64.0 (16/25)	Improving	0.067	0.177	0.543
C	38.5 (5/13)	60.0 (6/10)	30.0 (3/10)	Declining	1.000	0.370	0.414
D	38.7 (12/31)	52.2 (35/67)	63.6 (7/11)	Improving	0.180	0.533	0.278
E	50.0 (12/24)	78.3 (18/23)	18.8 (3/16)	Declining	0.056	<0.001	0.069
F	55.2 (16/29)	70.0 (14/20)	72.7 (16/22)	Improving	0.250	1.0000	0.377

^a^The without-fail rate was the proportion of patients at the facility who received all of the seven processes of care for which they were eligible

^**b**^Sustainment classification was defined a priori on the basis of the absolute change in the WFR between the active implementation period and the sustainment period: “No change” if within ±2.0%; “Improving” if >2.0% increase; and “Declining” if 2.0% decrease

Few statistically significant differences in patient characteristics were observed between PREVENT and control sites (Supplemental Table A; see footnote regarding statistical significance). During the sustainment period differences between implementation and control sites included: the rate of being admitted (versus discharged from the Emergency Department; 79.8% for PREVENT, 63.9% for controls), Hispanic ethnicity (12.1% for PREVENT, 4.9% for controls), neurology visit within 30 days post-discharge (53.2% for PREVENT, 43.4% for controls), and the CHADVASC score (3.5 for PREVENT and 3.2 for controls; all p<0.05).

### Quantitative results: quality of care during the sustainment period

**Fig. 1:**
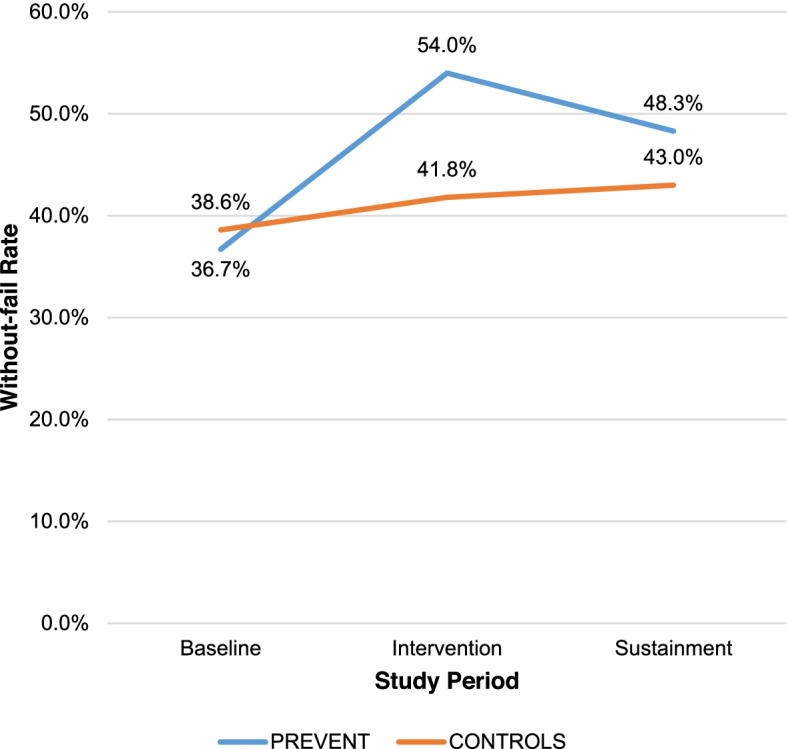
Change in TIA Quality of Care: Intervention versus matched control sites

The observed (unadjusted) without-fail rate improved at PREVENT sites from 36.7% (baseline) to 54.0% (active implementation) [21] and settled to 48.3% during sustainment. At control sites, the without-fail rate improved from 38.6% (baseline) to 41.8% (active implementation) [21] and continued at 43.0% during sustainment (Figure 1). The without-fail rate varied both across sites and time periods (Tables 1 and 2). Some PREVENT implementation sites demonstrated sustainment in terms of quality of care: the absolute change in the without-fail rate improved by ≥2% at 3 sites, declined by ≥2% at two sites, and remained unchanged at one site during sustainment. It was this heterogeneity in sustainment of quality of care across the six active PREVENT sites that allowed for qualitative examination of factors that were associated with sustained quality of care (see below).

**Table 2:** Comparing change over time at PREVENT sites versus matched control sites^a^

Quality of Care	Control Sites			PREVENT Sites			Adjusted Comparison Baseline versus Implementation Periods			Adjusted Comparison Baseline versus Sustainment Periods		
	Baseline Period ***N***=973	Implementation Period ***N***=968	Sustainment Period ***N***=758	Baseline Period ***N***=162	Implementation Period ***N***=189	Sustainment Period ***N***=124	Control	PREVENT	Interaction	Control	PREVENT	Interaction
	% (Pass/Eligible)	% (Pass/Eligible)	% (Pass/Eligible)	% (Pass/Eligible)	% (Pass/Eligible)	% (Pass/Eligible)	OR (95% CI)	OR (95% CI)	P-value	OR (95% CI)	OR (95% CI)	*P*-value
Anticoagulation for Atrial Fibrillation	74.8 (95/127)	75.2 (106/141)	82.6 (90/109)	63.3 (19/30)	100.0 (27/27)	76.0 (19/25)	1.4 (0.7, 2.8)	23.6 (1.2, 467.4)	0.072	2.0 (1.0, 4.2)	2.7 (0.7, 10.8)	0.704
Antithrombotics	94.2 (746/792)	93.9 (750/799)	95.7 (581/607)	97.9 (139/142)	96.4 (159/165)	97.1 (102/105)	1.5 (0.9, 2.7)	0.3 (0.1, 1.7)	0.078	2.4 (1.3, 4.6)	0.5 (0.1, 3.8)	0.151
Brain Imaging	94.4 (828/877)	94.7 (807/852)	91.6 (621/678)	93.7 (148/158)	98.3 (173/176)	94.8 (109/115)	1.0 (0.7, 1.6)	3.9 (1.02, 15.0)	0.059	0.7 (0.5, 1.04)	1.2 (0.4, 3.5)	0.325
Carotid Artery Imaging	75.5 (641/849)	77.6 (653/841)	78.8 (524/665)	76.8 (119/155)	85.0 (147/173)	86.7 (98/113)	1.0 (0.8, 1.3)	1.8 (1.0, 3.5)	0.095	1.3 (1.0, 1.7)	2.0 (1.0, 4.2)	0.224
High/Moderate Potency Statin	65.7 (478/727)	70.1 (508/725)	69.3 (386/557)	67.6 (92/136)	81.6 (124/152)	84.4 (76/90)	1.1 (0.9, 1.5)	2.0 (1.0, 3.8)	0.125	1.1 (0.8, 1.4)	2.1 (1.0, 4.7)	0.103
Hypertension Control	75.5 (468/620)	74.7 (485/649)	78.1 (395/506)	77.5 (93/120)	82.3 (102/124)	73.3 (63/86)	1.0 (0.7, 1.3)	1.2 (0.6, 2.3)	0.665	1.2 (0.9, 1.6)	0.8 (0.4, 1.6)	0.317
Neurology Consultation	73.8 (627/850)	80.1 (675/843)	82.9 (551/665)	66.5 (103/155)	79.8 (138/173)	82.3 (93/113)	1.4 (1.0, 1.8)	2.1 (1.2, 3.7)	0.181	1.4 (1.0, 1.9)	2.3 (1.2, 4.5)	0.171
**Mean Without-Fail Rate**	38.6 (345/893)	41.8 (363/869)	43.0 (293/681)	36.7 (58/158)	54.0 (95/176)	48.3 (56/116)	1.1 (0.9, 1.3)	2.0 (1.2, 3.3)	0.018	1.2 (1.0, 1.5)	1.6 (0.9, 2.7)	0.403
**Mean Consolidated Rate**	0.80 (0.21)	0.83 (0.20)	0.83 (0.19)	0.80 (0.20)	0.88 (0.16)	0.87 (0.15)	0.01 (-0.003, 0.03)	0.07 (0.03, 0.11)	0.008	0.02 (0.007, 0.04)	0.05 (0.01, 1.0)	0.165

^a^OR refers to adjusted odds ratios

In adjusted analyses, the without-fail rate improved at PREVENT sites compared with control sites during active implementation [21], however, no statistically significant difference in quality was identified during sustainment (Table 2; Supplemental Table B). In sensitivity analyses, the point estimates were very similar to those obtained from the main analysis after removing the site with the shortest sustainment duration (Supplemental Table C).

During sustainment, neither the consolidated measure of quality nor any of the seven individual processes of care were statistically different in intervention versus control sites after risk-adjustment (Table 2).

### Qualitative comparative case analyses

The qualitative comparative case analysis is summarized in Table 3. The only factor that was necessary and sufficient for improving quality of care during the sustainability phase was the presence of a champion with plans for sustainment at the end of active implementation (sites B, D, F; Table 3).

“The new nurse practitioner, one of his jobs is going to be to get the (TIA/Stroke) dashboard set up, and he will make the phone calls and hopefully see the patients while they’re in the hospital. Because he only has five and a half days of clinic. So every day he has a half of a day of clinic and a half day of other duties that he’s doing, and so he can see the patients in the hospital, make sure that everything is getting done, and then he can ensure follow-up, and he’s going to start monitoring like the first wave of stroke data with our quality person.”[Sustainment Site-F_5]

**Table 3:** PREVENT sustainment qualitative case comparison analysis by site

Facility	Sustainment planning at end of active implementation by champions^d^	Motivation to sustain^a^	Champion active during sustainment^b^	Planning during sustainment^b^	Goals & Feedback during sustainment^b^	Folded PREVENT into Champion’s scope of practice^c^	Reflecting & Evaluating on performance data^b^	Competing priorities stated^c^	Champion attended Community of Practice in sustainment^c^	Leadership engagement during sustainment^c^	Sustainment Classification^**e**^
A	A	High	+2	0	+1	A	+1	P	P	P	No Change
B	P	High	+2	0	0	P	+1	P	A	P	Improving
C	A	Low	0	0	0	A	0	P	A	A	Declining
D	P	High	+2	+1	0	A	+2	A	N/A	P	Improving
E	A	Low	0	0	0	A	0	P	A	A	Declining
F	P	High	+2	+1	+1	P	+1	A	P	P	Improving

^a^Motivation to sustain PREVENT’s rating was based on qualitative responses and classified as “high motivation” if participants discussed positive motivation to sustain and “low motivation” if the absence, lack of, or no motivation was noted

^b^Sustainment strategies were rated at the site level by the evaluation team as: +2 present moderate to strong; +1 present weak to moderate; 0 absent

^c^Thematic analysis of PREVENT site teams (clinical providers) semi-structured interviews during sustainment results were coded as: P if present; A if absent; or N/A if not applicable (i.e., Community of Practice sessions ended after all teams completed active implementation)

^d^Sustainment planning at end of active implementation was based upon PREVENT site teams semi-structured interviews after 12 months of active implementation but prior to sustainment]

^**e**^Sustainment classification was defined a priori on the basis of the absolute change in the without-fail rate between the active implementation period and the sustainment period: “No change” if within ±2.0%; “Improving” if >2.0% increase; and “Declining” if 2.0% decrease

Four factors were sufficient for declining quality of care during the sustainability period (sites C and E; Table 3): low motivation to sustain, 0 champion active during sustainment, 0 reflecting and evaluating on performance data, and absence of leadership engagement during sustainment.

Responses to interview questions about motivation to sustain PREVENT varied. The sites (C and E) with declining quality of care lacked robust motivation.

“I’ll be honest. When I found out about it [PREVENT], it kind of got thrown in my lap at the last minute...” [C_12M_1_SV]

In contrast, team members at sites with improving quality of care (B, D, F) described their motivation to sustain PREVENT.

“Because I think it’s the right thing to do and was even more convinced of it after participating in PREVENT.”[Sustainment Site-F_2]

“Well, I think that it’s just that, my interest in stroke. I mean I'm a stroke physician, and after strokes happen, there is almost very little that you can do…You know prevention in general is the biggest way that you can impact stroke in the nation and in our veterans is to stop it before it starts, and so if we treat the TIA seriously, then that they’re not going to come back with another stroke.”[Sustainment Site-F_5]

“I mean honestly I think it’s just, like I said it’s an easily done intervention that makes an impact on patients. And at this point it’s just part of my work…like I said, as part of being sort of responsible for stroke…”[Sustainment Site-B_2]

“…you know I’m a Veteran myself, so always improving healthcare is definitely a passion of mine.”[Sustainment Site-D_1]

The effect of the practice of reflecting and evaluating on sustainment could be viewed either in terms of its absence (a score of 0) at the two sites with declining quality of care during sustainment (sites C and E) or any presence (a score of +1 or +2) at the other sites (A, B, D, F). Four champions (A, B, D, F) continued to review, reflect, and evaluate their quality performance during the sustainability period; the site D team continued to reflect and evaluate as a team (+2 score).

“Yeah, and now he’s [Clinical Champion] meeting with our General Practice Manager with the data and he’s getting more proficient with that as well. Like there’s like a Sparks report. And he’s seeing like you know how well they are, and like what quadrant they fall in. So he’s learning more about that. So he still has lots of questions on all that data piece there; but he’s starting to get really kind of more proficient with that. So I mean we’re pretty productive, like we’re in the quadrant 2 for our site in Neurology, but you know I’m trying to push them forward to like hey…how do we get to quadrant 1…it’s like you know in the case where we do need a business case to move forward like okay we’re not, like what’s in it for Neurology, right? It’s that if we, they need more, right? And they’re not capturing active workload, then what’s going to happen, right? So we’re not going to be able to hire. We’re trying to figure out what we can do to be better in terms of–sort of like why are we seeing, why are people using the Mission X so they’ll understand kind of what all that means.”[Sustainment Site-D_2]

In another example, site B’s champion utilized local tools to monitor quality performance to track facility performance and communicate with clinicians about individual patients.

“By having the dashboard, it helps me capture patients that I myself would not necessarily have known about, and can therefore – so what I’m doing is when I see them there, then I am writing a note in the chart that was a templated note that our CAC developed on the metrics. And it says goal is met or it shows me how the goal was not met. And then I can flag the patient's primary care doctor; or if they happen to already have a neurologist for a different reason or something I also copy the neurologist on the issue that still needs to be addressed.”[Sustainment Site-B_2]

The effect of how active champions were during sustainment could similarly be viewed either in terms of absence of champion activity (a score of 0) at the two sites with declining quality of care during sustainment (sites C and E) or presence (scores of +2) at the other sites. The site E champion explicitly stated that he did not consider himself to be a PREVENT champion during sustainability. Site C team discussed how their low volume did not warrant continued attention during sustainability. Neither champion at sites C or E were aware of what their PREVENT teammates were doing in terms of PREVENT protocols during sustainability.

“I guess that I haven’t been as hands on with like the sustainability portion of the study. I guess that our site hasn't...We haven't like met since I guess that we graduated. So I'm not sure of exactly the progress of everything like that.”[Sustainment Site-C_3].”

“So basically the net effect was that the group as a system for checks wasn't as…involved anymore…it just kind of went to the individual members to see what their responsibilities were.” [Sustainment Site-E_1]

In contrast, the champion at site B (a site with improving quality during sustainment) was very active during the sustainability period; they monitored performance data and sent electronic messages to clinicians who missed an opportunity to provide a process of care to an eligible patient and pointed out how to improve that clinical process.

“So in a few cases, the primary doctors have actually written me back and said oh thank you for letting me know, we’ll schedule the patient in sooner or I’ll call them to change their dosing. Sometimes I’m not hearing directly but I’ve heard at times they’ll say ‘oh yes, I saw you’ve been writing those notes on patients in the charts that were, once they’re in the outpatient stage.’ So presumably it’s helping them act on it.”[Sustainment Site-B_2]

The other factors were heterogeneous in terms of their relationship with sustaining; planning during sustainment, having goals with feedback, folding PREVENT into the champion’s scope of practice, competing priorities, and champion’s attendance at the PREVENT Community of Practice were not consistently associated with sustainment positively or negatively. For example, champions at sites B and F (both with improving quality of care during sustainment) folded PREVENT into their scope of practices. During sustainability interviews, they stated that there was no more PREVENT program; rather, this is now their usual practice which included the champions operating within their scope of practice.

“…we changed the process, and so it’s not something that I have to monitor every day…So I think that the culture, and also we [PREVENT Team] changed the culture…But I do think that we made sustainable process and culture changes that this helped.”[Sustainment Site-F_5]

In contrast, at site A (which had no change in quality of care during sustainment), the champion discussed how a different champion was needed to sustain PREVENT, one who had the scope of practice to incorporate PREVENT over the long term.

“For a while I was sending out reports on a daily basis like you know, this is what we’re running and this is where we need to be, just trying to get like some facility ownership, someone to say okay, well we need to help pull those patients out of the ED, it’s like a facility responsibility. And I think one of the providers in the ER called it my public shaming report, but it was just trying to get some accountability and to get people to understand like this is a facility priority, and some quality improvement that we should be working together on, and it’s not just like an ER responsibility. So I report on that monthly now. We’ve improved our time now and we’re meeting target for admission delay now. But it’s been probably a yearlong process to kind of get physicians on board and involved. And even though now that’s been added to fail data, and you know it’s a facility quality improvement program, and it’s still data that’s very much collected, reported on, and driven by Nursing. With our stroke metrics, it’s the same situation. Unfortunately it’s like pulling teeth to try to get even people to even get like physician review of like policies and algorithms when it comes to appropriate stroke care. So, and like I’m not trying to complain, I’m just saying that it’s something that could definitely be improved upon here. But as far as like provider ownership or providers like pushing the protocol, I would say we sort of struggle in that area.”[Sustainment Site-A_1]

Although several of the sites described the presence of competing priorities, both of the sites with declining quality of care during sustainability (sites C and E) described the issue of competing priorities.

“So, it’s like a new project comes up, and we focus on that, and then it’s like on to the next project. On to the next project, and if we don’t sustain it, which it is more difficult, then we’ll probably start back at square one unfortunately.”[Sustainment Site-C_3]

### Qualitative results: factors associated with sustaining care quality

We interviewed 19 key stakeholders during the sustainment interviews across the six PREVENT teams: A – 2; B – 2; C – 3; D – 3; E – 3; F – 6. We have previous published a description of our PREVENT participants [42]. The Sustainment sample was a subsample of PREVENT active implementation per the teams’ recommendations.

The qualitative analyses identified two core activities that promoted sustainment of quality of care: (1) integrating processes of care into routine operations; and (2) reflecting and evaluating on performance data to plan quality improvement activities or respond to changes in quality. Two key challenges to sustainment were identified: (1) difficulties using performance data to inform quality improvement; and (2) competing demands from new facility quality priorities. Illustrative quotations are presented below with tags providing the interview timing and site label (consistent with Table 1).

### Promoting sustainment: Integrating processes of care into routine operations

Sites with improvements in care quality during sustainment embedded PREVENT activities within the fabric of routine workflow. For example, two sites included TIA quality of care as an ongoing topic into their existing stroke team meetings. Three sites provided regular updates to facility leadership about quality of TIA care within the context of regularly scheduled meetings:

“…it’s not so much that people are thinking of it as part of PREVENT, it’s just on that’s just what we do for patients.”[Sustainment Site-B_2]

And from a second site which improved during sustainment:

“…we changed the process, and so it’s not something that I have to monitor every day.”[Sustainment Site-F_5]

Several interviewees described automatization of processes such that they no longer required active attention or work.

“I think the biggest impact was…allowing things to be able to be somewhat on autopilot because there’s little that the clinicians have to do other than implement it or rather admit the patient. You know it flows very consistently with the existing process.”[Sustainment Site-F_2]

“It was pretty well hard-wired into our practices before we graduated the program. As we went through it was minor tweaks and changes so it’s already a regular deep-seated process for us.”[Sustainment Site-F_3]

**Promoting sustainment: Examining performance data regularly to identify and respond to changes in quality**

The sites that improved or maintained quality of care during sustainment used performance data to check on their status during the sustainment period.

“It was good to see the trending of our data points…when we had our group meetings and you know we would discuss those numbers…”[12 months Site-A_1]

At one site, the champion used the data to engage front-line staff:

“…sometimes it’s surprising like oh I didn’t know we were like this this last time I looked down and I looked at the Primary Care 30-day visit, and I was wondering oh wow, I didn’t know that was kind of lower than the national average for that…that kind of has given me something to think about to bring to like you know Neurology or like the hospitalist folks that I deal with on different kind of projects.”[12 months Site-D_1]

Quality of care data at the facility level allowed champions to identify patterns that might have been “invisible” to them (e.g., care provided by other services):

“By having the dashboard, it helps me capture patients that I myself would not necessarily have known about.”[Sustainment Site-B_2]

In contrast, team members at sites with declining quality of care during sustainment reported that they believed that PREVENT was integrated into existing structures or policies at the facility but did not actively examine performance data during sustainment, and were therefore, unaware of evidence of decrements in performance during sustainment.

“I think that all of the things that we did do are still in place today. So that’s really good.”[Sustainment Site-C_2]

“I haven’t been as hands on with like the sustainability portion of the study. Since we haven't met as a team since we graduated, I can't really speak on how well that we’ve sustained. I still think that we’re all taking care of TIA patients and things like that, but as far as looking over the seven criteria as a team together, we haven't done that…Probably just not paying attention I guess.[Sustainment Site-C_3],

“…the changes that we have implemented have been sustained. So the same changes have been maintained throughout the facility after the graduation.”[Sustainment Site-E_2]

### Barriers to sustainment: Difficulties using performance data to inform quality improvement

Respondents at sites that improved during sustainment identified two challenges related to using performance data for quality improvement: the difficulty of interpreting pass or fail rates in the setting of low patient volume, and coding problems that made it difficult to discern if without-fail rates reflected genuine issues with patient care or were spurious.

“…the biggest barrier that we’ve faced is just coincidence in numbers. This year, our TIA numbers have actually been less than in past years…The most that we’re able to do is review the cases and see…where things went wrong and what we could do to fix it…it’s really hard to identify patterns when the pattern is like we have one patient to review. Like sometimes it’s a little easier when you see like hey, if we had like ten patients, we’d be like all right. Eight were fine, but the pattern of the people who did have issues was this. But I think that that’s what’s really been the toughest part.”[12 month Site-C_1]

### Concerns about miscoding made data more challenging to interpret:

“…there have been a few cases where I think that either their TIA was mislabeled and we didn't like push or emphasize it strongly enough…”[12 month Site-E_1].

Some site team members identified relatively infrequent coding issues which nonetheless influenced their performance data. For example, a patient coded as having atrial fibrillation and identified as failing the anticoagulation metric may not actually have had atrial fibrillation, and therefore it was appropriate not to prescribe an anticoagulant. Given that the without-fail metric was based on administrative data, coding errors required chart review for identification, and working with facility coders for remediation.

### Barriers to sustainment: Competing demands

Respondents across sites identified the problem of competing demands on time as the major threat to sustainment.

“I mean I think that it’s a great program. You know. To come together with like a common goal and actually see how good the results can be. I think that it’s an awesome program. I just wish that there was a way for us to I guess keep everything going. I mean I know that we were still keeping things going, but it’s like they’ll come to us and say we’re trying to improve CHF care, and then we’ll do that, and then it’s like going on to the next project.”[Sustainment Site-E_3]

“I haven't been able to do anything with the sustainability portion, like I said, just because there are a ton of other projects, and I don’t want it to sound like that I'm giving an excuse, but there is literally like a million other things that management focuses on or that comes up in the ED.”[12 month Site-C_3]

## Discussion

The PREVENT program successfully improved quality of care during active implementation, but performance after active implementation was heterogeneous across sites. This site-to-site variability during sustainment provided an opportunity to examine factors which promoted sustainment as well as barriers to sustainment. Overall, quality of care at active implementation sites was not statistically different from quality at control sites during the sustainability period after adjustment [43].

The two factors that were most consistently and robustly associated with sustainment were integration of intervention processes into routine care and instituting practices for reviewing and reflecting upon performance data. Both activities appear to be necessary but not sufficient for successful sustainment. For example, sites where team members reported perceptions that processes were embedded into routine practice but did not report ongoing evaluation of performance data declined in performance during sustainment, despite team members’ impressions that they were doing very well in terms of care quality. In these latter cases, the success of the active implementation phase led the team members to erroneously believe that their work was completed and that they no longer needed to monitor their performance data. Our prospective approach—which allowed us to examine quantitative change in performance and qualitative interview data—highlighted the perils among clinical teams during sustainment who perceived their work to be complete after achieving quality improvement during active implementation. These results align with the literature that has identified the need for ongoing review of performance data as key to maintaining quality; the qualitative results, especially the quotations, provide real-life reminders of the emotional and cognitive perspectives that may serve as barriers to sustainment [2, 6].

Our comparative case analyses revealed that site champions for TIA/Stroke care who were highly motivated to sustain PREVENT at the local facility, folded PREVENT into their regular scope of practice, engaged with local leadership, and reflected and evaluated their local performance data by which they made sustainment plans and goals were more likely to sustain PREVENT compared to sites with an absence of motivation, inactive champions who did not reflect and evaluate on performance data and who lacked leadership engagement. The site champions who were successful in sustaining and improving acute stroke/TIA quality continued to engage in quality improvement practices (reflecting and evaluating upon their local data; setting goals with feedback, planning activities) that were similarly associated with successful active implementation [22]. These champions continued to engage with local leadership to implement plans, report on quality performance and provide leadership with assistance with quality improvement in other clinical areas by invitation. These results reinforce the PREVENT’s facilitation team’s investment in teaching and promoting reflecting and evaluating on data, setting goals, and feedback and planning. Future work may identify efficient approaches (e.g., playbooks) and packaging to promote implementation and sustainment.

Although the PREVENT intervention included some elements focused on sustainment; they were insufficient to ensure sustainment across all sites. Our qualitative results align with the report by Lennox, et al [44] that sought to identify factors that promoted initial success as distinct from factors which promoted sustainment (e.g., monitoring for feedback and learning) [44]. A systematic review identified 40 constructs related to sustainability including items related to resources (which includes staff and time), demonstrating effectiveness, and monitoring progress over time [45]. Future research should identify how implementation strategies differ from and interact with sustainment strategies, and when sustainment strategies need to be initiated. For example, some PREVENT teams focused on building structures that worked to deliver desired care (usually dependent on a single staff person) but delayed integrating those structures within the fabric of the facility’s routine workflow—despite recognition that this was needed for sustainment. Future studies should explicitly identify barriers to sustainment early during implementation and then evaluate approaches to overcoming those barriers.

The primary difference between active implementation and sustainment was the withdrawal of external facilitation. External facilitation is recognized as an important implementation strategy [46]. Our finding that performance improvement gains made during active implementation did not persist during sustainment supports the importance of external facilitation to quality improvement [22, 39]. PREVENT facilitation activities were comparable to other implementation trials [39, 47]. However, in addition to supporting action plan activities, PREVENT facilitation promoted reflecting and evaluating on performance data, setting and modifying goal, and planning activities for implementation and sought to enhance quality improvement skills. Unlike approaches used in other studies [48], we did not identify an internal facilitator at participating sites and transfer external facilitation tasks to the local site. We did seek to include systems redesign staff in each of the participating sites teams; however at only one site did this person become a PREVENT clinical champion. One key role that the external facilitators played during active implementation was to alert site teams to declines in performance [24]; future studies should examine whether technological approaches to implementing a Learning Healthcare System might support sustainment (e.g., automated alerts when performance changes to trigger teams to action). External facilitation has been demonstrated to be resource intensive [38]; future studies should examine whether the intensity and domains of external facilitation may be modified during sustainment while still maintaining quality improvement achievements.

A strength of the study design was the inclusion of matched control sites. If we had only examined changes in care quality among PREVENT sites, then we may have concluded that the PREVENT program successfully improved quality during active implementation (36.7% to 54.0%); and that quality diminished somewhat but remained higher than baseline during sustainment (48.3%). By examining performance in the context of control sites, we understand that care quality at intervention sites was similar to control sites during sustainment (48.3% versus 43.0%). Future stepped-wedge trial designs should consider augmentation with controls to assess temporal trends both during active implementation and sustainment.

Several study limitations merit description. First, because of the stepped-wedge design, the last site enrolled had the shortest sustainment duration. Although this site maintained its sample size, changes in practices or quality over time might have not be observed. Sensitivity analyses which involved removing this site provided results which were similar to the main findings suggesting that the overall results were not driven by this one site. Prior research has suggested that fixed sustainment periods designated in trial designs may be arbitrary and may not be of adequate duration to identify sites which were initially unsuccessful with sustainment but which improve over time [49]. Second, PREVENT was implemented within VA facilities, which may limit its generalizability. Third, there may be some diagnostic uncertainty when making a diagnosis of TIA [50]. Therefore, changes in quality that were observed in this study may differ from studies of conditions with less clinical uncertainty (e.g., myocardial infarction). Fourth, the qualitative results were limited in that they represented only six sites. Fifth, heterogeneity in eligibility for the seven processes of care may have influenced the without-fail rate across facilities. For example, sites with higher proportions of patients receiving hospice care or early mortality would have fewer patients eligible for quality assessment. We reported adjusted and unadjusted results to illustrate how differences in patient characteristics may have influenced the assessment of quality; very few differences in point estimates were observed. Sixth, the collaborative conferences and PREVENT hub were available to sites throughout the project. Future studies should evaluate whether a decrement in quality might have occurred after all resources were withdrawn. Seventh, the rationale for sustaining the implementation of evidence-based practices rests in the associated improvement in patient outcomes. Although the without-fail rate metric of care quality is based on guideline recommended processes of care (e.g., prescription of statins, antithrombotics, blood pressure control), future studies should examine effects on patient outcomes such as recurrent stroke or death. Finally, the intervention included multiple integrated components and we are unable to isolate and estimate the unique effects of each specific element.

Overall, the PREVENT program enhanced quality of care during active implementation but not during sustainment. Heterogeneity in performance during sustainment across facilities provided an opportunity to identify factors associated with sustainment suggesting that facilities seeking to embody the Learning Healthcare System’s core values should harness the combined power of staff and data systems to embed quality improvement within routine care and establish systems for reviewing and reflecting upon performance data [51].

## Supplementary Information

**Additional file 1.**

**Additional file 2.**

## Data Availability

The quality of care data must remain behind the US Department of Veterans Affairs (VA) firewall. The de-identified qualitative data must remain on VA servers. Interested investigators are encouraged to contact the corresponding author for new analyses of the existing data.
