# Supplementary material for: The Perils of a “My Work Here is Done” perspective: a mixed methods evaluation of sustainment of an evidence-based intervention for transient ischemic attack
Source: BMC Health Serv Res. 2022 Jul 4;22:857. doi: 10.1186/s12913-022-08207-8 (PMC9254423; doi:10.1186/s12913-022-08207-8)
Supplement: Supplementary file 1 — Additional file 1. [file 12913_2022_8207_MOESM1_ESM.docx]

**SUPPLEMENTAL Table A. Baseline Characteristics: PREVENT and Control Sites Across Three Time Periods**

| **Patient Characteristics** | **Baseline** | | **Implementation** | | **Sustainment** | |
| --- | --- | --- | --- | --- | --- | --- |
|  | **PREVENT**  (N=162 patients)  (N=6 sites) | **Control**  (N=973 patients)  (N=36 sites) | **PREVENT**  (N=189 patients)  (N=6 sites) | **Control**  (N=968 patients)  (N=36 sites) | **PREVENT**  (N=124 patients)  (N=6 sites) | **Control**  (N=758 patients)  (N=36 sites) |
| **Index Event** |  |  |  |  |  |  |
| ED-Only for index event | 22.8 (37) | 35.5 (345) | 19.0 (36) | 32.0 (310) | 20.2 (25) | 36.1 (274) |
| Admitted for index event | 77.2 (125) | 64.5 (628) | 81.0 (153) | 68.0 (658) | 79.8 (99) | 63.9 (484) |
| Weekday presentation | 80.2 (130) | 80.4 (782) | 75.7 (143) | 78.4 (759) | 82.3 (102) | 76.9 (583) |
| Left AMA | 2.5 (4) | 3.9 (38) | 4.8 (9) | 5.4 (52) | 5.6 (7) | 4.7 (36) |
| **Demographics** |  |  |  |  |  |  |
| Age (years): mean ± standard deviation (SD) | 69.8 (11.2) | 71.7 (11.3) | 68.8 (11.3) | 71.4 (10.9) | 71.5 (10.9) | 71.3 (11.2) |
| Median (interquartile range [IQR]) | 69.0 (63.0-76.0) | 71.0 (65.0-80.0) | 69.0 (61.0-75.0) | 71.0 (65.0-78.0) | 72.0 (66.0-77.0) | 72.0 (65.0-79.0) |
| Male sex | 95.1 (154) | 94.6 (920) | 95.8 (181) | 94.4 (914) | 95.2 (118) | 95.0 (720) |
| Race^§^ |  |  |  |  |  |  |
| White | 71.6 (116) | 79.1 (770) | 65.6 (124) | 75.1 (727) | 70.2 (87) | 71.0 (538) |
| Black | 22.8 (37) | 14.9 (145) | 26.5 (50) | 19.0 (184) | 23.4 (29) | 24.5 (186) |
| Asian | 0.0 (0) | 0.5 (5) | 1.1 (2) | 0.9 (9) | 0.8 (1) | 0.3 (2) |
| Other | 0.6 (1) | 0.4 (4) | 0.5 (1) | 0.8 (8) | 0.8 (1) | 0.3 (2) |
| Unknown | 4.9 (8) | 5.0 (49) | 6.3 (12) | 4.1 (40) | 4.8 (6) | 4.0 (30) |
| Hispanic ethnicity | 11.1 (18) | 4.6 (45) | 8.5 (16) | 6.0 (58) | 12.1 (15) | 4.9 (37) |
| **Past Medical History** |  |  |  |  |  |  |
| TIA in prior 30 days | 4.3 (7) | 5.8 (56) | 5.8 (11) | 3.9 (38) | 4 (5) | 3.6 (27) |
| Stroke in prior 30 days | 4.3 (7) | 6.7 (65) | 5.3 (10) | 5.9 (57) | 4 (5) | 6.1 (46) |
| Diabetes mellitus | 48.1 (78) | 41.5 (404) | 41.3 (78) | 42.5 (411) | 43.5 (54) | 40.6 (308) |
| Atrial fibrillation | 21.6 (35) | 17.6 (171) | 16.9 (32) | 18.4 (178) | 21 (26) | 19.3 (146) |
| Myocardial infarction | 8.6 (14) | 7.3 (71) | 5.8 (11) | 8.2 (79) | 10.5 (13) | 8.6 (65) |
| Congestive heart failure | 13.6 (22) | 16.8 (163) | 11.1 (21) | 16.9 (164) | 21.8 (27) | 17.9 (136) |
| Carotid endarterectomy or stent | 0.6 (1) | 1.1 (11) | 0.0 (0) | 0.6 (6) | 1.6 (2) | 0.5 (4) |
| Chronic obstructive pulmonary disease (COPD) | 16.7 (27) | 21.3 (207) | 19.0 (36) | 20.9 (202) | 22.6 (28) | 23.0 (174) |
| Peripheral arterial disease | 12.3 (20) | 17.0 (165) | 15.3 (29) | 15.8 (153) | 19.4 (24) | 15.7 (119) |
| Dementia | 9.9 (16) | 8.3 (81) | 6.3 (12) | 6.5 (63) | 9.7 (12) | 6.5 (49) |
| Chronic kidney disease | 17.9 (29) | 16.4 (160) | 14.3 (27) | 18.1 (175) | 21 (26) | 18.5 (140) |
| Dialysis | 3.7 (6) | 1.3 (13) | 0.5 (1) | 2.2 (21) | 1.6 (2) | 1.7 (13) |
| Cancer | 9.3 (15) | 12.6 (123) | 11.1 (21) | 10.3 (100) | 15.3 (19) | 10.8 (82) |
| Hypertension | 79.6 (129) | 73.6 (716) | 80.4 (152) | 77.1 (746) | 80.6 (100) | 78.0 (591) |
| Hyperlipidemia | 67.9 (110) | 60.0 (584) | 63.5 (120) | 63.0 (610) | 61.3 (76) | 64.1 (486) |

**SUPPLEMENTAL Table A. (continued)**

| **Patient Characteristics** | **Baseline** | | **Implementation** | | **Sustainment** | |
| --- | --- | --- | --- | --- | --- | --- |
|  | **PREVENT**  (N=162 patients)  (N=6 sites) | **Control**  (N=973 patients)  (N=36 sites) | **PREVENT**  (N=189 patients)  (N=6 sites) | **Control**  (N=968 patients)  (N=36 sites) | **PREVENT**  (N=124 patients)  (N=6 sites) | **Control**  (N=758 patients)  (N=36 sites) |
| Speech deficit | 8.0 (13) | 4.5 (44) | 6.3 (12) | 5.9 (57) | 7.3 (9) | 5.3 (40) |
| Motor deficit, hemiplegia | 15.4 (25) | 13.2 (128) | 21.2 (40) | 16.8 (163) | 22.6 (28) | 17.0 (129) |
| Sleep apnea | 21.0 (34) | 18.2 (177) | 23.3 (44) | 24.5 (237) | 27.4 (34) | 23.0 (174) |
| Alcohol dependence | 8.6 (14) | 7.4 (72) | 11.1 (21) | 8.0 (77) | 8.1 (10) | 7.7 (58) |
| Depression | 27.8 (45) | 21.5 (209) | 22.2 (42) | 23.7 (229) | 26.6 (33) | 25.7 (195) |
| History of venous thromboembolism* | 7.4 (12) | 3.1 (30) | 3.7 (7) | 3.9 (38) | 1.6 (2) | 3.2 (24) |
| Intracranial hemorrhage | 8.0 (13) | 5.9 (57) | 5.3 (10) | 5.4 (52) | 2.4 (3) | 2.9 (22) |
| Gastrointestinal bleeding | 1.2 (2) | 0.7 (7) | 0.5 (1) | 0.5 (5) | 1.6 (2) | 0.7 (5) |
| Migraine | 4.9 (8) | 2.6 (25) | 4.8 (9) | 2.9 (28) | 4 (5) | 4.4 (33) |
| Medications prior to index event: |  |  |  |  |  |  |
| Antihypertensives | 87.7 (142) | 84.1 (818) | 84.7 (160) | 83.7 (810) | 87.1 (108) | 84.2 (638) |
| Statin | 83.3 (135) | 80.7 (785) | 89.4 (169) | 83.5 (808) | 90.3 (112) | 83.6 (634) |
| Aspirin | 81.5 (132) | 75.3 (733) | 78.8 (149) | 71.0 (687) | 75.8 (94) | 71.8 (544) |
| Warfarin | 6.8 (11) | 12.8 (125) | 9.0 (17) | 8.9 (86) | 8.1 (10) | 8.2 (62) |
| CHADVASC* | 3.3 (1.4) | 3.3 (1.5) | 3.1 (1.3)^†^ | 3.3 (1.5) | 3.5 (1.4)^†^ | 3.2 (1.4) |
| HASBLED* | 2.2 (1.1) | 2.2 (1.0) | 2.1 (1.0) | 2.2 (1.1) | 2.3 (1.1) | 2.3 (1.0) |
| Charlson: mean ± SD | 2.8 (2.8) | 2.9 (2.7) | 2.7 (2.8) | 2.9 (2.7) | 3.1 (2.8) | 3.0 (2.8) |
| Median (IQR) | 2.0 (0.0-4.0) | 2.0 (1.0-4.0) | 2.0 (0.0-4.0) | 2.0 (1.0-4.0) | 2.5 (1.0-5.0) | 2.0 (1.0-4.0) |
| Smoker | 27.2 (44) | 27.2 (265) | 32.3 (61) | 26.8 (259) | 21.8 (27) | 26.3 (199) |
| Palliative care, hospice | 1.9 (3) | 4.5 (44) | 1.6 (3) | 3.2 (31) | 2.4 (3) | 2.6 (20) |
| **Laboratory and Vital Signs** |  |  |  |  |  |  |
| APACHE:* mean ± standard deviation | 10.2 (7.4) | 9.5 (6.7) | 9.2 (6.1) | 10.2 (6.9) | 10.5 (6.8) | 10.1 (6.7) |
| First Systolic blood pressure (mm Hg): |  |  |  |  |  |  |
| Mean Systolic (SD) | 145.3 (24.1) | 147.7 (25.8) | 142.8 (25.2) | 148.5 (25.8) | 149.0 (26.1) | 148.0 (24.7) |
| Median Systolic (IQR) | 144.0 (128.0-162.0) | 147.0 (129.0-165.0) | 142.0 (125.0-159.0) | 148.0 (130.0-164.0) | 149.0 (128.0-166.0) | 147.0 (130.0-164.0) |
| First Diastolic blood pressure (mm Hg): |  |  |  |  |  |  |
| Mean Diastolic (SD) | 81.0 (14.6) | 80.3 (13.1) | 80.2 (14.4) | 81.2 (14.4) | 82.0 (13.6) | 81.8 (14.5) |
| Median Diastolic (IQR) | 80.0 (72.0-90.0) | 80.0 (71.0-89.0) | 79.0 (71.0-90.0) | 81.0 (71.0-90.0) | 81.0 (72.0-91.0) | 80.0 (72.0-91.0) |

**SUPPLEMENTAL Table A. (continued)**

| **Patient Characteristics** | **Baseline** | | **Implementation** | | **Sustainment** | |
| --- | --- | --- | --- | --- | --- | --- |
|  | **PREVENT**  (N=162 patients)  (N=6 sites) | **Control**  (N=973 patients)  (N=36 sites) | **PREVENT**  (N=189 patients)  (N=6 sites) | **Control**  (N=968 patients)  (N=36 sites) | **PREVENT**  (N=124 patients)  (N=6 sites) | **Control**  (N=758 patients)  (N=36 sites) |
| Average Systolic blood pressure 90 days post discharge (mm Hg): |  |  |  |  |  |  |
| Mean Systolic (SD) | 130.3 (15.6) | 131.4 (16.4) | 127.5 (14.1) | 131.2 (15.8) | 129.6 (14.4) | 129.7 (15.5) |
| Median Systolic (IQR) | 130.0 (120.0-138.9) | 130.4 (120.0-141.0) | 126.6 (118.0-136.3) | 130.0 (121.3-139.5) | 130.0 (120.3-137.0) | 129.5 (119.0-139.0) |
| Average Diastolic blood pressure 90 days post discharge (mm Hg): |  |  |  |  |  |  |
| Mean Diastolic (SD) | 74.6 (9.3) | 74.5 (9.3) | 73.1 (9.5) | 73.7 (9.7) | 74.8 (8.3) | 74.0 (9.4) |
| Median Diastolic (IQR) | 75.0 (69.0-80.0) | 74.5 (68.7-80.0) | 73.0 (67.0-80.0) | 74.0 (67.0-80.0) | 73.8 (68.7-79.5) | 74.0 (67.5-80.0) |
| **Healthcare Utilization** |  |  |  |  |  |  |
| Any inpatient admission in year prior to index event | 34.0 (55) | 31.1 (303) | 22.8 (43) | 28.9 (280) | 30.6 (38) | 31.9 (242) |
| Any Emergency Department visit in year prior to index event | 54.9 (89) | 61.5 (598) | 55.0 (104) | 60.6 (587) | 61.3 (76) | 62.8 (476) |
| Primary care visit in 30-days post-discharge | 62.3 (101) | 58.5 (569) | 63.0 (119) | 59.9 (580) | 62.9 (78) | 62.5 (474) |
| Primary care visit in 90-days post-discharge | 86.4 (140) | 79.7 (775) | 82.0 (155) | 81.9 (793) | 79.8 (99) | 82.1 (622) |
| Neurology visit in 30-days post-discharge | 18.5 (30) | 20.2 (197) | 15.9 (30) | 20.6 (199) | 33.1 (41) | 21.9 (166) |
| Neurology visit in 90-days post-discharge | 45.1 (73) | 39.0 (379) | 43.4 (82) | 42.5 (411) | 53.2 (66) | 43.4 (329) |

*Venous thromboembolism refers to (deep vein thrombosis or pulmonary embolism); CHADVASC is a score that is used to predicts risk of thromboembolism among patients with atrial fibrillation; the HASBLED score provides an assessment of bleeding risk; and the Acute Physiology and Chronic Health Evaluation (APACHE) score is a measure of physiological disease severity.

A statistically significant difference (p<0.05) in patient characteristics at PREVENT sites was noted across time for median age, congestive heart failure, CHADVASC, and neurology visits at 30-days post-discharge.

^§^A statistically significant difference (p<0.05) in patient characteristics between PREVENT implementation sites versus control sites was noted as follows. During the baseline period differences between implementation and control sites included the rate of being admitted (versus discharged from the Emergency Department [ED]5), Hispanic ethnicity, dialysis, venous thromboembolism, warfarin, and age. During the implementation period, differences between implementation and control sites included: the rate of being admitted (versus discharged from the ED), statin, aspirin, age, HASBLED, and mean systolic blood pressure. During the sustainment period differences between implementation and control sites included: the rate of being admitted (versus discharged from the ED), Hispanic ethnicity, neurology visit within 30 days post-discharge, and the CHADVASC score.

**SUPPLEMENT Table B. Comparing Change Over Time at PREVENT Sites Versus Matched Control Sites – Baseline vs. Sustainment**

| **Quality of Care** | **Control Sites** | | **PREVENT Sites** | | **Unadjusted** | | | **Adjusted** | | |
| --- | --- | --- | --- | --- | --- | --- | --- | --- | --- | --- |
|  | **Baseline** | **Sustainment** | **Baseline** | **Sustainment** | **Control** | **PREVENT** | **Interaction** | **Control** | **PREVENT** | **Interaction** |
|  | %  (Pass/Eligible) | %  (Pass/Eligible) | % (Pass/Eligible) | %  (Pass/Eligible) | **OR**  **(95% CI)** | **OR**  **(95% CI)** | **P-value** | **OR**  **(95% CI)** | **OR**  **(95% CI)** | **P-value** |
| Anticoagulation for Atrial Fibrillation | 74.8  (95/127) | 82.6  (90/109) | 63.3  (19/30) | 76.0  (19/25) | 1.6  (0.8, 3.0) | 1.8  (0.5, 5.7) | 0.876 | 2.0  (1.0, 4.2) | 2.7  (0.7, 10.8) | 0.704 |
| Antithrombotics | 94.2  (746/792) | 95.7  (581/607) | 97.9  (139/142) | 97.1  (102/105) | 1.4  (0.8, 2.3) | 0.8  (0.1, 3.8) | 0.485 | 2.4  (1.3, 4.6) | 0.5  (0.1, 3.8) | 0.151 |
| Brain Imaging | 94.4  (828/877) | 91.6  (621/678) | 93.7  (148/158) | 94.8  (109/115) | 0.7  (0.5, 1.01) | 1.2  (0.4, 3.5) | 0.311 | 0.7  (0.5, 1.04) | 1.2  (0.4, 3.5) | 0.325 |
| Carotid Artery Imaging | 75.5  (641/849) | 78.8  (524/665) | 76.8  (119/155) | 86.7  (98/113) | 1.2  (0.9, 1.5) | 2.1  (1.1, 4.1) | 0.116 | 1.3  (1.0, 1.7) | 2.0  (1.0, 4.2) | 0.224 |
| High/Moderate Potency Statin | 65.7  (478/727) | 69.3  (386/557) | 67.6  (92/136) | 84.4  (76/90) | 1.1  (0.9, 1.5) | 2.7  (1.3, 5.3) | 0.022 | 1.1  (0.8, 1.4) | 2.1  (1.0, 4.7) | 0.103 |
| Hypertension Control | 75.5  (468/620) | 78.1  (395/506) | 77.5  (93/120) | 73.3  (63/86) | 1.2  (0.9, 1.5) | 0.8  (0.4, 1.6) | 0.356 | 1.2  (0.9, 1.6) | 0.8  (0.4, 1.6) | 0.317 |
| Neurology Consultation | 73.8  (627/850) | 82.9  (551/665) | 66.5  (103/155) | 82.3  (93/113) | 1.4  (1.1, 1.9) | 2.5  (1.3, 4.8) | 0.110 | 1.4  (1.0, 1.9) | 2.3  (1.2, 4.5) | 0.171 |
| **Mean Without-Fail Rate** | 38.6  (345/893) | 43.0  (293/681) | 36.7  (58/158) | 48.3  (56/116) | 1.1  (0.9, 1.4) | 1.6  (1.0, 2.7) | 0.167 | 1.2  (1.0, 1.5) | 1.6  (0.9, 2.7) | 0.403 |
| **Mean (SD) Consolidated Rate** | 0.80 (0.21) | 0.83 (0.19) | 0.80 (0.20) | 0.87 (0.15) | 0.02  (-0.001, 0.04) | 0.07  (0.03, 0.12) | 0.035 | 0.02  (0.007, 0.04) | 0.05  (0.01, 1.0) | 0.165 |

**Supplementary Table C. Comparing Change Over Time at PREVENT Sites Versus Matched Control Sites: Excluding the Wave-3 Site with the Shortest Sustainability Period**

| **Quality of Care** | **Control Sites** | | | **PREVENT Sites** | | | **Adjusted Comparison**  **Baseline versus Implementation** | | | **Adjusted Comparison**  **Baseline versus Sustainment** | | |
| --- | --- | --- | --- | --- | --- | --- | --- | --- | --- | --- | --- | --- |
|  | **Baseline** | **Implementation** | **Sustainment** | **Baseline** | **Implementation** | **Sustainment** | **Control** | **PREVENT** | **Interaction** | **Control** | **PREVENT** | **Interaction** |
|  | % (Pass/Eligible) | %  (Pass/Eligible) | %  (Pass/Eligible) | % (Pass/Eligible) | %  (Pass/Eligible) | %  (Pass/Eligible) | OR  (95% CI) | OR  (95% CI) | P-value | OR  (95% CI) | OR  (95% CI) | P-value |
| Anticoagulation for Atrial Fibrillation | 72.3  (73/101) | 76.3  (87/114) | 83.7  (82/98) | 65.2  (15/23) | 100.0  (21/21) | 73.9  (17/23) | 1.6  (0.8, 3.2) | 20.5  (0.98, 429.2) | 0.105 | 2.4  (1.1, 5.4) | 2.8  (0.6, 12.7) | 0.861 |
| Antithrombotics | 94.3 (611/648) | 93.8  (605/645) | 95.6 (523/547) | 98.2 (112/114) | 96.1  (98/102) | 96.8  (91/94) | 1.5  (0.8, 2.8) | 0.1  (0.2, 1.1) | 0.030 | 2.3  (1.1, 4.7) | 0.3  (0.1, 2.9) | 0.091 |
| Brain Imaging | 94.4 (670/710) | 94.6  (653/690) | 91.8 (557/607) | 93.7 (119/127) | 98.2  (107/109) | 94.2  (98/104) | 1.0  (0.6, 1.6) | 3.5  (0.7, 17.2) | 0.143 | 0.7  (0.5, 1.1) | 1.0  (0.4, 3.1) | 0.548 |
| Carotid Artery Imaging | 75.4 (525/696) | 77.7  (529/681) | 78.8 (471/598) | 75.8  (94/124) | 86.9  (93/107) | 87.3  (89/102) | 1.0  (0.8, 1.4) | 1.9  (0.9, 4.2) | 0.123 | 1.2  (0.9, 1.7) | 2.2  (1.0, 4.8) | 0.184 |
| High/Moderate Potency Statin | 66.9 (397/593) | 69.4  (411/592) | 71.1 (355/499) | 64.5  (69/107) | 83.7  (82/98) | 84.0  (68/81) | 1.0  (0.8, 1.4) | 2.7  (1.2, 6.0) | 0.023 | 1.1  (0.8, 1.5) | 2.4  (1.0, 5.6) | 0.076 |
| Hypertension Control | 76.8 (394/513) | 74.7  (393/526) | 76.8 (348/453) | 75.0  (72/96) | 77.1  (64/83) | 71.8  (56/78) | 0.9  (0.7, 1.3) | 1.0  (0.7, 1.4) | 0.848 | 1.0  (0.7, 1.4) | 0.8  (0.4, 1.7) | 0.585 |
| Neurology Consultation | 78.2 (545/697) | 84.8  (578/682) | 84.6 (506/598) | 66.1  (82/124) | 81.3  (87/107) | 83.3  (85/102) | 1.4  (1.0, 1.9) | 2.0  (1.0, 4.1) | 0.328 | 1.3  (0.9, 1.8) | 2.6  (1.3, 5.4) | 0.088 |
| **Mean Without-Fail Rate** | 40.1 (291/726) | 43.3  (304/702) | 43.4 (265/610) | 36.2  (46/127) | 55.0  (60/109) | 46.7  (49/105) | 1.0  (0.8, 1.3) | 2.1  (1.2, 3.7) | 0.028 | 1.2  (0.9, 1.5) | 1.4  (0.8, 2.6) | 0.495 |
| **Mean Consolidated Rate** | 0.81 (0.21) | 0.83 (0.20) | 0.84 (0.18) | 0.79 (0.21) | 0.88 (0.16) | 0.87 (0.15) | 0.01  (-0.01, 0.03) | 0.07  (0.02, 0.11) | 0.013 | 0.02  (0.003, 0.04) | 0.06  (0.01, 1.0) | 0.160 |

**Supplementary Table D. Multivariable Modeling for the Without-Fail Rate**

| **Variable** | **OR (95% CI)** | **P-value** |
| --- | --- | --- |
| **Wave** |  |  |
| 2 Sites | 0.56 (0.14, 2.31) | 0.284 |
| 3 Sites | 0.71 (0.17, 2.93) | 0.502 |
| 1 Sites (reference) | 1.00 |  |
| Age (years) | 0.99 (0.98, 0.99) | 0.001 |
| History of TIA (outpatient encounter) | 2.63 (2.17, 3.18) | <0.001 |
| Hemiplegia | 1.23 (0.99, 1.54) | 0.068 |
| History of Dialysis | 0.98 (0.52, 1.86) | 0.959 |
| Amaurosis Fugax | 1.20 (0.80, 1.79) | 0.378 |
| HASBLED Score* | 1.04 (0.94, 1.14) | 0.468 |
| Palliative/Hospice Care | 41.29 (18.39, 92.73) | <0.001 |
| Aspirin | 1.45 (1.16, 1.80) | 0.001 |
| Warfarin | 1.90 (1.43, 2.53) | <0.001 |
| Statin | 3.85 (2.82, 5.26) | <0.001 |
| Clopidogrel | 1.44 (1.19, 1.73) | <0.001 |
| **Implementation * PREVENT** |  | 0.018 |
| Implementation vs. Baseline - Control Sites | 1.07 (0.86, 1.33) | 0.554 |
| Implementation vs. Baseline - PREVENT Sites | 2.04 (1.25, 3.32) | 0.004 |
| **Sustainment * PREVENT** |  | 0.403 |
| Sustainment vs. Baseline - Control Sites | 1.22 (0.97, 1.54) | 0.091 |
| Sustainment vs. Baseline - PREVENT Sites | 1.57 (0.91, 2.70) | 0.104 |

*The HASBLED score provides an assessment of bleeding risk.

**Supplementary Table E. Multivariable Modeling for the Consolidated Measure of Quality of Care**

| **Variable** | **Estimate (95% CI)** | **P-value** |
| --- | --- | --- |
| **Wave** |  |  |
| 2 Sites | -0.04 (-0.14, 0.06) | 0.443 |
| 3 Sites | -0.03 (-0.13, 0.07) | 0.559 |
| 1 Sites (reference) | 0.00 |  |
| Age (years) | 0.00 (0.00, 0.00) | 0.001 |
| History of TIA (outpatient encounter) | 0.11 (0.10, 0.12) | <0.001 |
| Hemiplegia | 0.03 (0.01, 0.05) | 0.001 |
| History of Dialysis | -0.09 (-0.14, -0.04) | <0.001 |
| Amaurosis Fugax | 0.02 (-0.01, 0.05) | 0.295 |
| HASBLED Score* | 0.00 (-0.01, 0.01) | 0.770 |
| Palliative/Hospice Care | 0.13 (0.09, 0.16) | <0.001 |
| Aspirin | 0.04 (0.03, 0.06) | <0.001 |
| Warfarin | 0.06 (0.04, 0.08) | <0.001 |
| Statin | 0.11 (0.09, 0.13) | <0.001 |
| Clopidogrel | 0.03 (0.01, 0.04) | <0.001 |
| **Implementation * PREVENT** |  | 0.008 |
| Implementation vs. Baseline - Control Sites | 0.01 (-0.003, 0.03) | 0.102 |
| Implementation vs. Baseline - PREVENT Sites | 0.07 (0.03, 0.11) | <0.001 |
| **Sustainment * PREVENT** |  | 0.165 |
| Sustainment vs. Baseline - Control Sites | 0.02 (0.01, 0.04) | 0.006 |
| Sustainment vs. Baseline - PREVENT Sites | 0.06 (0.01, 0.10) | 0.008 |

*****The HASBLED score provides an assessment of bleeding risk.

**Supplementary Table F. Multivariable Modeling for the Neurology Consultation Process of Care**

| **Variable** | **OR (95% CI)** | **P-value** |
| --- | --- | --- |
| **Wave** |  |  |
| 2 Sites | 0.23 (0.02, 3.42) | 0.184 |
| 3 Sites | 0.24 (0.02, 3.48) | 0.187 |
| 1 Sites (reference) | 1.00 |  |
| Age (years) | 0.98 (0.97, 0.99) | <0.001 |
| History of Transient Ischemic Attack (outpatient encounter) | 2.61 (2.06, 3.31) | <0.001 |
| Hemiplegia | 2.13 (1.51, 3.00) | <0.001 |
| Amaurosis Fugax | 1.16 (0.66, 2.04) | 0.595 |
| Statin | 1.08 (0.80, 1.46) | 0.613 |
| Clopidogrel | 1.19 (0.93, 1.52) | 0.166 |
| **Implementation * PREVENT** |  | 0.181 |
| Implementation vs. Baseline - Control Sites | 1.37 (1.03, 1.82) | 0.030 |
| Implementation vs. Baseline - PREVENT Sites | 2.11 (1.20, 3.73) | 0.010 |
| **Sustainment * PREVENT** |  | 0.171 |
| Sustainment vs. Baseline - Control Sites | 1.41 (1.03, 1.91) | 0.030 |
| Sustainment vs. Baseline - PREVENT Sites | 2.34 (1.21, 4.52) | 0.012 |

**Supplementary Table G. Multivariable Modeling for the Hypertension Control Process of Care**

| **Variable** | **OR (95% CI)** | **P-value** |
| --- | --- | --- |
| **Wave** |  |  |
| 2 Sites | 1.01 (0.72, 1.42) | 0.961 |
| 3 Sites | 1.39 (0.97, 1.99) | 0.075 |
| 1 Sites (reference) | 1.00 |  |
| Age (years) | 0.99 (0.98, 1.00) | 0.283 |
| Systolic Blood Pressure | 0.97 (0.97, 0.98) | <0.001 |
| APACHE score* | 0.98 (0.96, 1.00) | 0.022 |
| History of Hypertension | 0.84 (0.61, 1.17) | 0.310 |
| History of Hyperlipidemia | 1.25 (0.98, 1.59) | 0.076 |
| History of Arrhythmia | 1.08 (0.76, 1.52) | 0.670 |
| Antihypertensive Medications | 0.61 (0.41, 0.89) | 0.011 |
| **Implementation * PREVENT** |  | 0.665 |
| Implementation vs. Baseline - Control Sites | 0.98 (0.75, 1.29) | 0.897 |
| Implementation vs. Baseline - PREVENT Sites | 1.15 (0.59, 2.27) | 0.678 |
| **Sustainment * PREVENT** |  | 0.317 |
| Sustainment vs. Baseline - Control Sites | 1.20 (0.89, 1.62) | 0.242 |
| Sustainment vs. Baseline - PREVENT Sites | 0.82 (0.41, 1.62) | 0.560 |

*The Acute Physiology and Chronic Health Evaluation (APACHE) score is a measure of physiological disease severity.

**Supplementary Table H. Multivariable Modeling for the High or Moderate Potency Statin Process of Care**

| **Variable** | **OR (95% CI)** | **P-value** |
| --- | --- | --- |
| **Wave** |  |  |
| 2 Sites | 0.88 (0.43, 1.81) | 0.622 |
| 3 Sites | 0.80 (0.39, 1.64) | 0.391 |
| 1 Sites (reference) | 1.00 |  |
| History of Transient Ischemic Attack (outpatient encounter) | 1.86 (1.49, 2.32) | <0.001 |
| History of Chronic Kidney Disease | 1.37 (1.00, 1.88) | 0.053 |
| History of Cirrhosis | 0.31 (0.13, 0.73) | 0.008 |
| History of Valvular Disease | 1.25 (0.85, 1.85) | 0.254 |
| Aspirin | 1.48 (1.15, 1.89) | 0.002 |
| Statin | 22.28 (15.54, 31.93) | <0.001 |
| Clopidogrel | 1.64 (1.28, 2.09) | <0.001 |
| **Implementation * PREVENT** |  | 0.125 |
| Implementation vs. Baseline - Control Sites | 1.14 (0.87, 1.49) | 0.353 |
| Implementation vs. Baseline - PREVENT Sites | 1.98 (1.03, 3.80) | 0.042 |
| **Sustainment * PREVENT** |  | 0.103 |
| Sustainment vs. Baseline - Control Sites | 1.06 (0.79, 1.41) | 0.707 |
| Sustainment vs. Baseline - PREVENT Sites | 2.13 (0.96, 4.71) | 0.062 |

**Supplementary Table I. Multivariable Modeling for the Carotid Artery Imaging Process of Care**

| **Variable** | **OR (95% CI)** | **P-value** |
| --- | --- | --- |
| **Wave** |  |  |
| 2 Sites | 0.65 (0.15, 2.73) | 0.405 |
| 3 Sites | 0.66 (0.16, 2.80) | 0.430 |
| 1 Sites (reference) | 1.00 |  |
| Age (years) | 0.99 (0.98, 1.00) | 0.008 |
| History of Transient Ischemic Attack (outpatient encounter) | 5.02 (4.04, 6.22) | <0.001 |
| History of Cerebral Amyloid Angiopathy | 0.49 (0.19, 1.28) | 0.147 |
| History of Chronic Kidney Disease | 0.86 (0.65, 1.13) | 0.279 |
| Hemiplegia | 1.42 (1.03, 1.95) | 0.030 |
| Amaurosis Fugax | 1.63 (0.94, 2.85) | 0.083 |
| Aspirin | 1.39 (1.11, 1.75) | 0.005 |
| Statin | 1.56 (1.20, 2.04) | 0.001 |
| Clopidogrel | 1.32 (1.04, 1.67) | 0.021 |
| **Implementation * PREVENT** |  | 0.095 |
| Implementation vs. Baseline - Control Sites | 1.03 (0.80, 1.33) | 0.812 |
| Implementation vs. Baseline - PREVENT Sites | 1.84 (0.98, 3.45) | 0.058 |
| **Sustainment * PREVENT** |  | 0.224 |
| Sustainment vs. Baseline - Control Sites | 1.25 (0.95, 1.65) | 0.107 |
| Sustainment vs. Baseline - PREVENT Sites | 2.02 (0.98, 4.15) | 0.055 |

**Supplementary Table J. Multivariable Modeling for the Brain Imaging Process of Care**

| **Variable** | **OR (95% CI)** | **P-value** |
| --- | --- | --- |
| **Wave** |  |  |
| 2 Sites | 1.46 (0.79, 2.71) | 0.223 |
| 3 Sites | 1.58 (0.83, 3.00) | 0.161 |
| 1 Sites (reference) | 1.00 |  |
| APACHE* score | 1.05 (1.02, 1.08) | <0.001 |
| History of Transient Ischemic Attack (outpatient encounter) | 2.30 (1.66, 3.20) | <0.001 |
| **Implementation * PREVENT** |  | 0.059 |
| Implementation vs. Baseline - Control Sites | 1.01 (0.66, 1.55) | 0.955 |
| Implementation vs. Baseline - PREVENT Sites | 3.93 (1.03, 15.04) | 0.045 |
| **Sustainment * PREVENT** |  | 0.325 |
| Sustainment vs. Baseline - Control Sites | 0.69 (0.46, 1.04) | 0.077 |
| Sustainment vs. Baseline - PREVENT Sites | 1.22 (0.42, 3.54) | 0.712 |

* The Acute Physiology and Chronic Health Evaluation (APACHE) score is a measure of physiological disease severity.

**Supplementary Table K. Multivariable Modeling for the Antithrombotics Process of Care**

| **Variable** | **OR (95% CI)** | **P-value** |
| --- | --- | --- |
| **Wave** |  |  |
| 2 Sites | 0.98 (0.56, 1.73) | 0.945 |
| 3 Sites | 1.56 (0.85, 2.86) | 0.146 |
| 1 Sites (reference) | 1.00 |  |
| History of Transient Ischemic Attack (outpatient encounter) | 13.99 (7.47, 26.20) | <0.001 |
| History of Atrial Fibrillation | 5.17 (2.03, 13.15) | 0.001 |
| Hemiplegia | 5.49 (1.22, 24.72) | 0.027 |
| Aspirin | 32.50 (18.99, 55.63) | <0.001 |
| Warfarin | 6.46 (1.80, 23.16) | 0.004 |
| Clopidogrel | 18.17 (6.32, 52.19) | <0.001 |
| **Implementation * PREVENT** |  | 0.078 |
| Implementation vs. Baseline - Control Sites | 1.53 (0.88, 2.68) | 0.135 |
| Implementation vs. Baseline - PREVENT Sites | 0.31 (0.06, 1.67) | 0.172 |
| **Sustainment * PREVENT** |  | 0.151 |
| Sustainment vs. Baseline - Control Sites | 2.43 (1.28, 4.61) | 0.007 |
| Sustainment vs. Baseline - PREVENT Sites | 0.54 (0.08, 3.83) | 0.538 |

**Supplementary Table L. Multivariable Modeling for the Anticoagulation Process of Care**

| **Variable** | **OR (95% CI)** | **P-value** |
| --- | --- | --- |
| **Wave** |  |  |
| 2 Sites | 0.92 (0.49, 1.73) | 0.791 |
| 3 Sites | 0.91 (0.48, 1.72) | 0.773 |
| 1 Sites (reference) | 1.00 |  |
| Age (years) | 0.96 (0.93, 0.98) | 0.002 |
| History of Atrial Fibrillation | 6.07 (2.96, 12.42) | <0.001 |
| History of Intracranial Hemorrhage | 0.70 (0.20, 2.43) | 0.571 |
| HASBLED* Score | 0.91 (0.70, 1.18) | 0.469 |
| Warfarin | 26.40 (7.50, 92.90) | <0.001 |
| **Implementation * PREVENT** |  | 0.072 |
| Implementation vs. Baseline - Control Sites | 1.43 (0.75, 2.75) | 0.278 |
| Implementation vs. Baseline - PREVENT Sites | 23.65 (1.20, 467.36) | 0.038 |
| **Sustainment * PREVENT** |  | 0.704 |
| Sustainment vs. Baseline - Control Sites | 2.00 (0.96, 4.17) | 0.063 |
| Sustainment vs. Baseline - PREVENT Sites | 2.71 (0.68, 10.83) | 0.157 |

*****The HASBLED score provides an assessment of bleeding risk.
